# Mixed Layer Depth Seasonality within the Coral Sea Based on Argo Data

**DOI:** 10.1371/journal.pone.0060985

**Published:** 2013-04-11

**Authors:** Jasmine B. D. Jaffrés

**Affiliations:** AIMS@JCU and School of Earth and Environmental Sciences, James Cook University, Townsville, Queensland, Australia; University of Vigo, Spain

## Abstract

The worldwide deployment of Argo floats has enabled much more detailed studies of global and regional seas over the last decade. Here, the seasonal variability of the mixed layer depth (MLD) within the Coral Sea was examined with CTD profiles from Argo floats. Multiple threshold values for both temperature and density have been employed to determine the most suitable threshold values for the Coral Sea. A threshold value of 0.04 kg/m^3^ for density and 0.2°C for temperature appear the most fitting for this region. Although MLD and isothermal layer depth (ILD) coincide quite well in most cases, the relatively common presence of temporary, non-seasonal barrier layers induces an ILD that is significantly deeper than the MLD. Consequently, an MLD estimation based on density is more appropriate. A distinct seasonality in the MLD is evident throughout the Coral Sea, but is generally more pronounced in higher southern latitudes (20–30°S). Salinity inversions are rare and mainly occur in the south-eastern Coral Sea, while barrier layers are more commonly associated with the north-eastern Coral Sea, a region characterised by high rainfall. The significance of regional currents is evident in the north-western Coral Sea, where temperature and ocean heat content is relatively low due to a northward moving boundary current. Shallow bathymetry, in turn, is linked to the absence of Argo data on the continental shelf and in the central Coral Sea.

## Introduction

Turbulent mixing leads to the formation and maintenance of a quasi-homogenous surface region of salinity, temperature and density that is generally interpreted as the ocean surface mixed layer [1]. This turbulent layer plays an important role in air-sea interactions through the flux and storage of heat, gases (e.g. CO_2_), and momentum [2]. The ocean mixed layer controls not only the depth over which the net surface heat flux is distributed [3], but also the depth from which nutrients are supplied to the surface [4], and the depth of the subsurface chlorophyll maxima [5]. The variability of the mixed layer is studied in terms of the mixed layer depth (MLD), a zone of very abrupt change in temperature and/or salinity, which defines the lower limit of the turbulent mixed layer [1].

**Figure 1:**
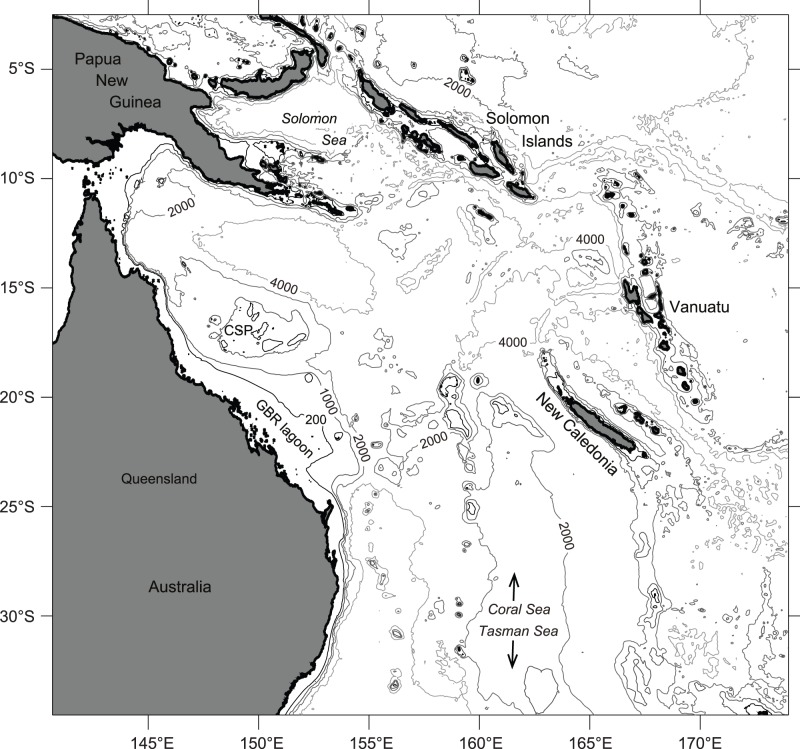
Bathymetry of the Coral Sea. CSP marks the shallow Coral Sea Plateau and GBR refers to the Great Barrier Reef. Isobaths for 200 m, 1000 m, 2000 m and 4000 m are displayed. The bathymetry was derived from DBDB2 (***Digital*** Bathymetric Data Base; an ongoing project of the U. S. Naval Research Laboratory).

The present MLD study focuses purely on the Coral Sea, with the conductivity-temperature-depth (CTD) profiles derived from Argo floats deposited within the Coral Sea or its vicinity since July 2001. The Coral Sea is a marginal sea located in the south-west Pacific off the northeast coast of Queensland, Australia (142°E), and bordered by the Solomon Islands and Papua New Guinea to the north (9°S), New Caledonia and Vanuatu to the east (170°E), and the Tasman Sea to the south (30°S) (Figure 1) [6]. The mean depth of the Coral Sea is around 2400 m, with the shallowest area being located on the continental coast of Queensland, Australia [6].

Since 1999, more than 3,000 Argo floats have been deployed across the world [7], with an expected lifespan of around 4–5 years for the individual Argo float, enabling detailed studies of the mixed layer behaviour and mid-depth circulation both in the global ocean and regional seas such as the Coral Sea. These freely drifting floats are parked at a preprogrammed pressure (usually 1000 dbar) from which, at predetermined intervals (typically 10 days), they will first descend to around 2000 m depth before rising to the surface, transmit data, and descend to the parking position [8]. Measurements of temperature and salinity are taken during the ascent with a CTD sensor module [9]. Additional parameters, including density and ocean heat content (OHC), can then be derived and utilised for further research topics, including mixed layers and currents.

**Figure 2:**
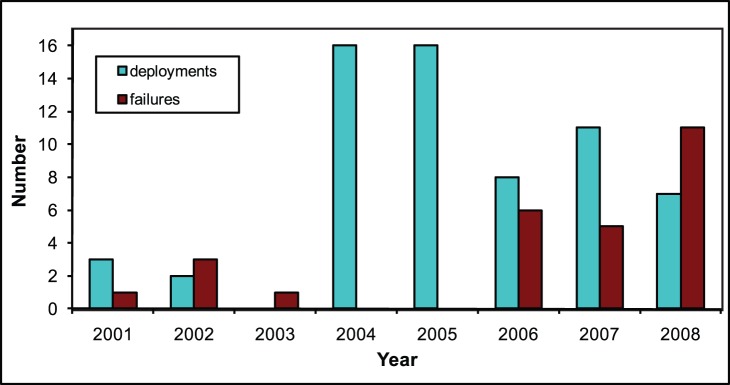
Yearly Argo deployments and failures. The number of new deployments and failures of Argo floats within or nearby the Coral Sea is shown per year.

**Figure 3:**
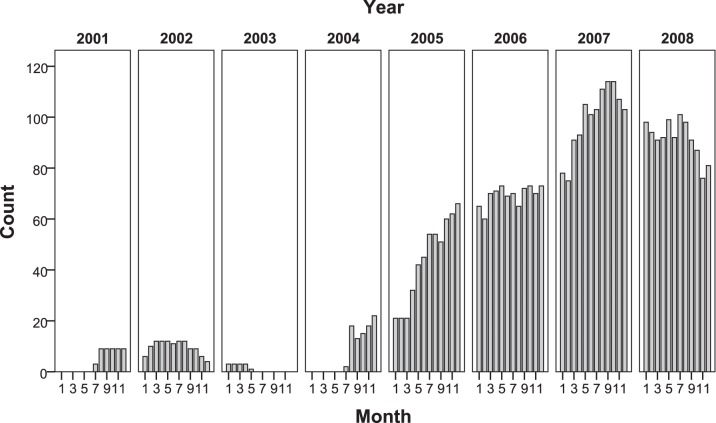
Total Argo CTD profile availability. The distribution of total number of Argo CTD profiles available within the Coral Sea prior to 2009 is displayed.

Numerous Argo floats were also deposited within the Coral Sea region, with 38 active floats as of 31 December 2008. Both the number and quality of Argo floats available improved dramatically over time. Prior to 2004, only five Argo floats were deployed within or near the Coral Sea (Figure 2); and even though a float’s life expectancy is currently around four years, these early floats had a maximum life of 15 months, resulting in a 1-year gap from mid-2003 to mid-2004 during which no Argo CTD measurements were taken (Figure 3). After mid-2004, the life of individual Coral Sea floats generally exceeded three years, with many floats deployed in the Coral Sea and its vicinity in 2004 and 2005 still being active at the end of 2008. The mean running time of Argo floats utilised in this project is 31.5 months, with the mean running time for newer floats being significantly higher (>30 months) than floats deployed before 2004 (8 months).

Previous studies incorporating MLD patterns within the Coral Sea have generally either focussed on a small area only [10], or have included the Coral Sea as part of a global study [11], [12]. The only study that has investigated the MLD variability within the Coral Sea in a little more detail was conducted by Condie and Dunn [13], in a regional study covering the Australasian seas. They applied threshold levels of ΔT = 0.4°C and ΔS = 0.3 psu (ΔS = change in salinity) for the entire region, thus not adapting the threshold method for the Coral Sea specifically. They presented interpolated maps of mixed layer seasonality, including their rms residuals, with most of their data derived from the World Ocean Database (WOD). Seasonally, their deepest Coral Sea MLDs are associated with winter in the south-west, while the shallowest MLDs occur during summer throughout the Coral Sea. The greatest variability is linked to the East Australian Current (EAC), a western boundary current moving southward along the east coast of Australia.

Here, multiple threshold values are applied for temperature and density to determine the most suitable threshold value for the Coral Sea. Furthermore, spatial and seasonal MLD variability based on the best MLD estimate are presented, with the data derived from Argo. The seasonal patterns of barrier layers and salinity inversions are also briefly discussed. Finally, the impact of currents and bathymetry on the Argo data distribution is explored.

## Methods

The threshold method is one of the most commonly employed techniques to derive the MLD. The threshold method identifies the depth at which the potential density changes by a fixed value (Δρ) relative to the value at a near-surface reference depth [11]. The reference depth is usually set at 10 m to avoid much of the strong diurnal variability in the top few meters of the ocean in equatorial regions [11]. Similarly, the isothermal layer depth (ILD) is defined as the depth, where the temperature has altered by a finite amount (ΔT) from the temperature at a reference depth [1]. Numerous studies have used the isothermal layer depth (ILD) to estimate the MLD due to the lack of available density profiles. However, the ILD and MLD do not always correspond. Consequently, taking into account that some future studies may only employ temperature profiles to investigate the MLD, both MLD and ILD are investigated here to determine the extent of agreement between them. Previous studies have determined that, on a global ocean scale, the optimal criterion value for ΔT is between 0.2°C [11] and 0.6°C [1], whereas a threshold value of 0.03 kg/m^3^ was considered most suitable for Δρ [11]. Nonetheless, the most accurate Δρ and ΔT values vary significantly both seasonally and regionally [1].

Here, four different threshold values have been evaluated for both the ILD (ΔT = ±0.1°C, ±0.15°C, ±0.2°C, ±0.25°C) and the MLD (Δρ = 0.025 kg/m^3^, 0.03 kg/m^3^, 0.035 kg/m^3^, 0.04 kg/m^3^), with the threshold reference depth set at 10 m, to estimate the most appropriate value for the Coral Sea region. In addition, similar to previous studies [14], all profiles were visually inspected to estimate a reference MLD (MLD_ref_) and ILD (ILD_ref_), taking into account both the alteration rate of density and temperature and their change from the reference depth (10 m). Density (ρ, kg/m^3^) was calculated using the One Atmosphere International Equation of State of Seawater, 1980 (see also [15]).

The Argo MLDs and ILDs obtained with the threshold method were compared closely with their respective MLD_ref_ and ILD_ref_, to determine the suitability of the threshold methods for the Coral Sea area. The comparison is performed by calculating the mean of paired absolute differences, bias (mean of differences), standard error (standard deviation divided by the sample size) and root-mean-square error (rmse). A positive (negative) bias represents an overestimation (underestimation) of the associated ILD or MLD. In addition, the skill score presented in Kara *et al*. [16] is computed to verify the most suitable threshold value (cf. Text S1 for details on the skill score calculation). CTD and density profiles in which the shallowest observation level is deeper than 10 m, or where the MLD is not well-developed, were excluded from the MLD analysis (cf. [17]). Additional MLDs, for which only the MLD_ref_ were determined, were derived from the World Ocean Database (WOD) to demonstrate the importance of the Argo project. WOD data covers to period of 1982 to 2003, overlapping with Argo during 2001–2003.

The important interplay between Argo data availability and both currents and bathymetry can be demonstrated by the resulting sea surface temperature (SST) and OHC patterns, or absence thereof. The SST is derived from the shallowest point of each Argo profile, provided its water depth is less than 10 m. The OHC has been obtained using the following formula:
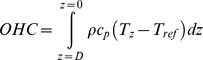
where *c_p_* is the specific heat capacity of seawater (4000 J kg^−1^ C^−1^), *ρ* is density, *T* is temperature, and *z* is the depth range of the OHC integration. The reference temperature *T_ref_* was arbitrarily set to zero, with the OHC equation then simplifying to:



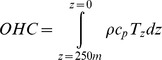



The OHC was computed to a depth of 250 m to ensure that both the maximum depth of surface oceanic mixing and a significant proportion of the subsurface currents are captured by the OHC calculation.

## Results and Discussion

### Impact of Coral Sea Bathymetry and Currents

**Figure 4:**
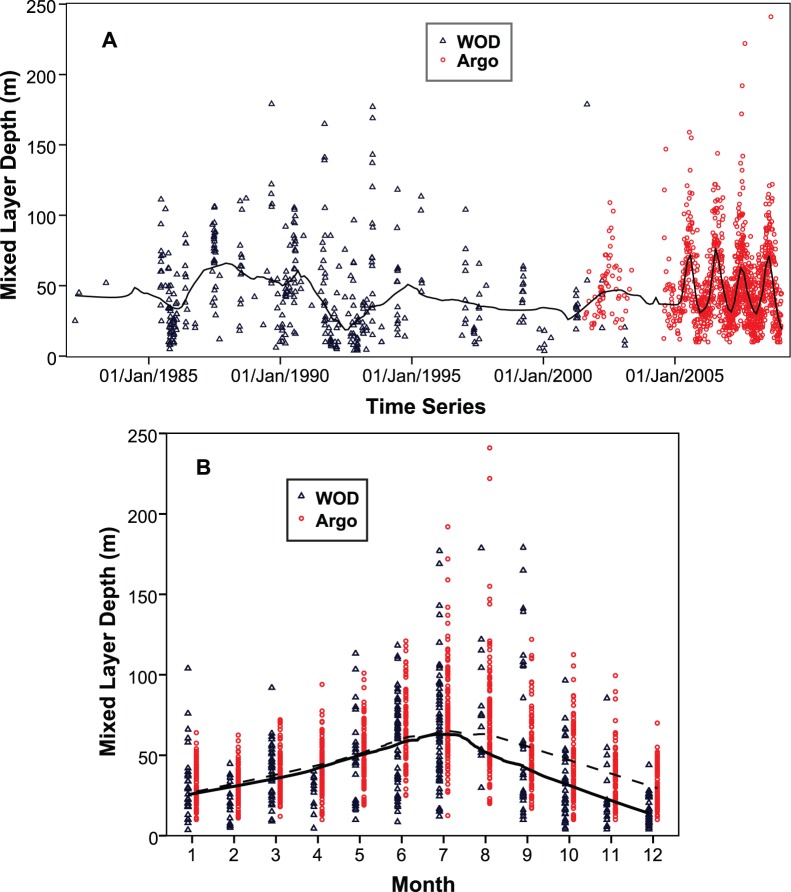
MLD seasonality and evolution of MLD data availability. A) Temporal trend of the mixed layer depth (MLD_ref_) within the Coral Sea since 1982. Pre-2001 MLDs were derived from CTD profiles of the World Ocean Database (WOD). The 2001–2003 period contains data from both WOD and Argo. B) Monthly observations of WOD and Argo mixed layer depths (MLD_ref_). For clarity, data in B) are shown in monthly groups, slightly offset, rather than in Julian days. The line of best fit (solid-black for WOD, dashed-grey for Argo) was created using the least-squared local regression (LOESS) method.

Since their introduction into the Coral Sea in 2001, Argo floats have produced at least as many CTD profiles as all shipboard CTD profiles stored in the WOD over the previous two decades. As a result, more detailed mixed layer analysis is now possible, with the Argo data displaying a much clearer seasonal signal than the pre-Argo period (Figure 4a). Due to their relatively deep parking depth, however, Argo floats generally can only remain operational when the water depth exceeds 2000 km, implying that no active floats operate within the shallow shelf regions.

**Figure 5:**
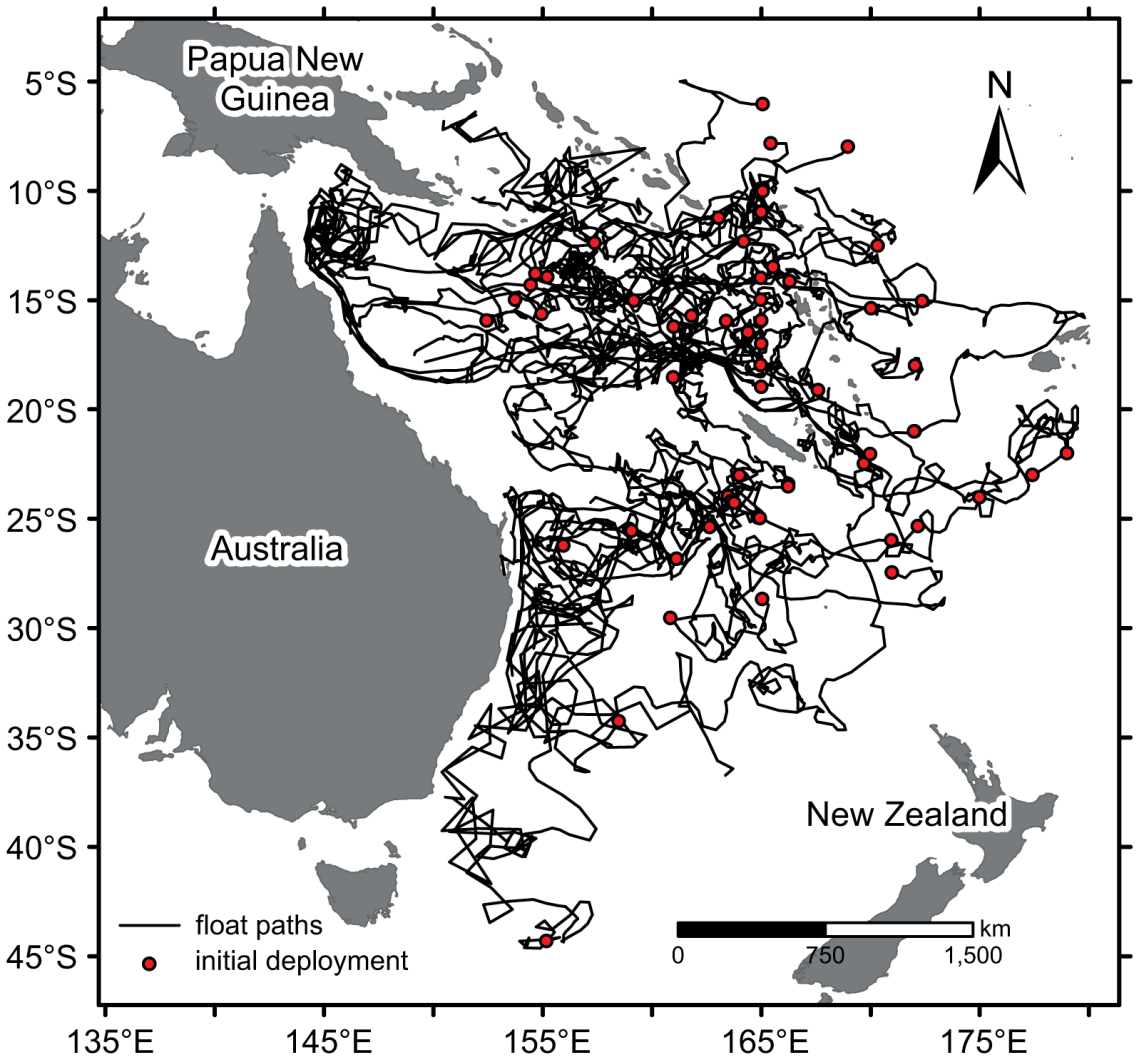
Paths of Argo floats deposited within or near the Coral Sea.

Most floats that entered or passed through the Coral Sea were either deposited in the eastern Coral Sea or in the West Pacific (Figure 5), and were carried deeper into the Coral Sea by the dominant currents (e.g. South Equatorial Current; [18]). Consequently, CTD profiles of the early years (2001–2004) are mainly from the eastern part of the Coral Sea.

Figure 5 shows the trajectories of the Argo floats until December 2008. There are several areas that display a greater density of float activity, corresponding to the following currents: (1) the North Vanuatu Jet (NVJ) and (2) the North Caledonian Jet (NCJ), both currents moving westward at ca. 14.0°S and 17.5°S, respectively [19]; and (3) the North Queensland Current (NQC) in the north-western Coral Sea, moving northwards along the Australian coast and clockwise along the southern coast of Papua New Guinea [20]. Following the westward transport by the NVJ and NCJ, many Argo floats were moved northwards into the Gulf of Papua by the NQC, while other floats stopped functioning after being stranded in shallow waters. Additional floats were deposited within the Tasman Sea, and moved northwards towards the southern Coral Sea border (the eddy-rich Tasman front region).

**Figure 6:**
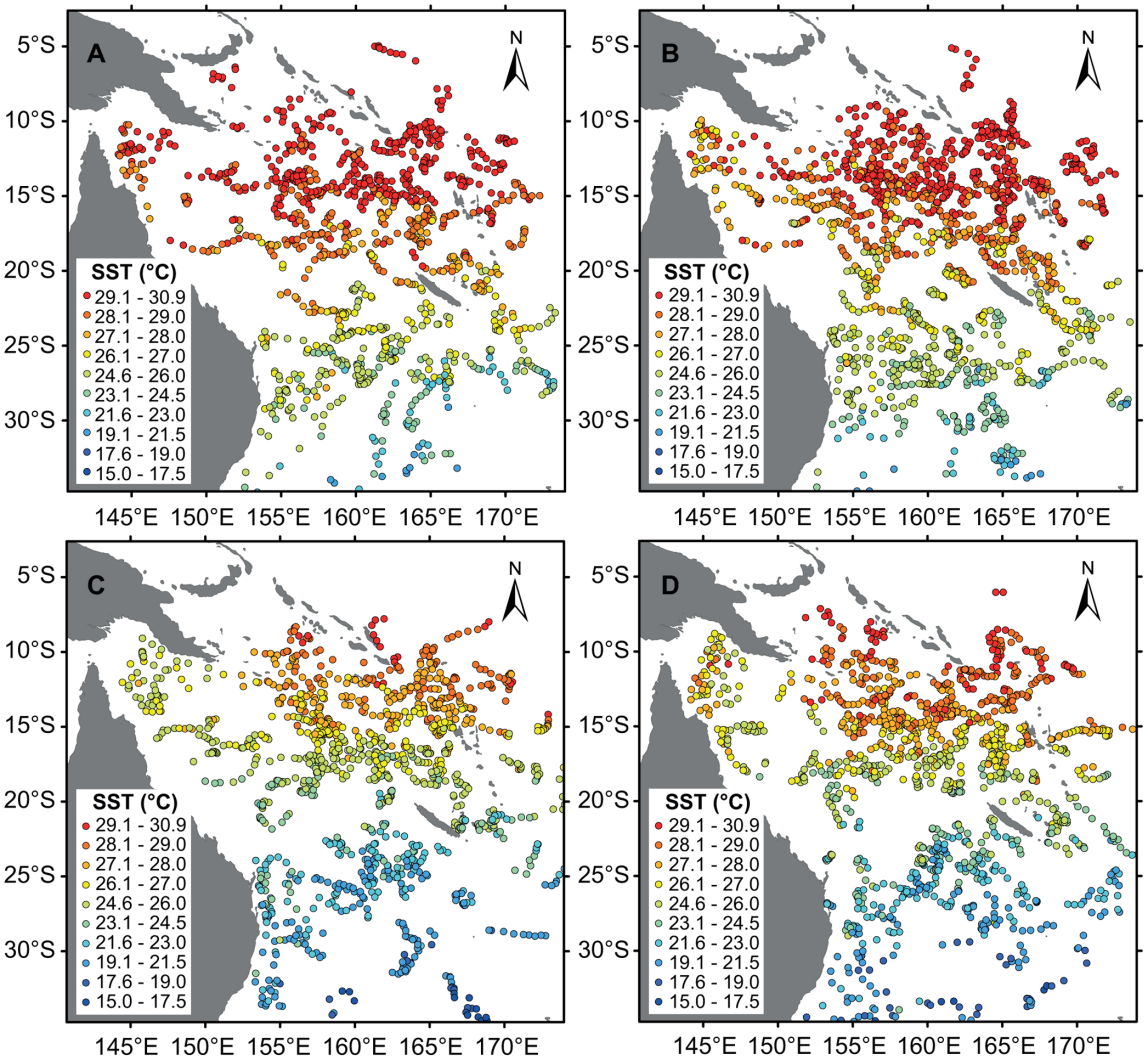
Seasonality of sea surface temperature (SST). Spatial distribution of SST during A) January–March, B) April–June, C) July–September and D) October–December. The SST data has been obtained from Argo floats and represent temperatures in the upper 10 m.

**Figure 7:**
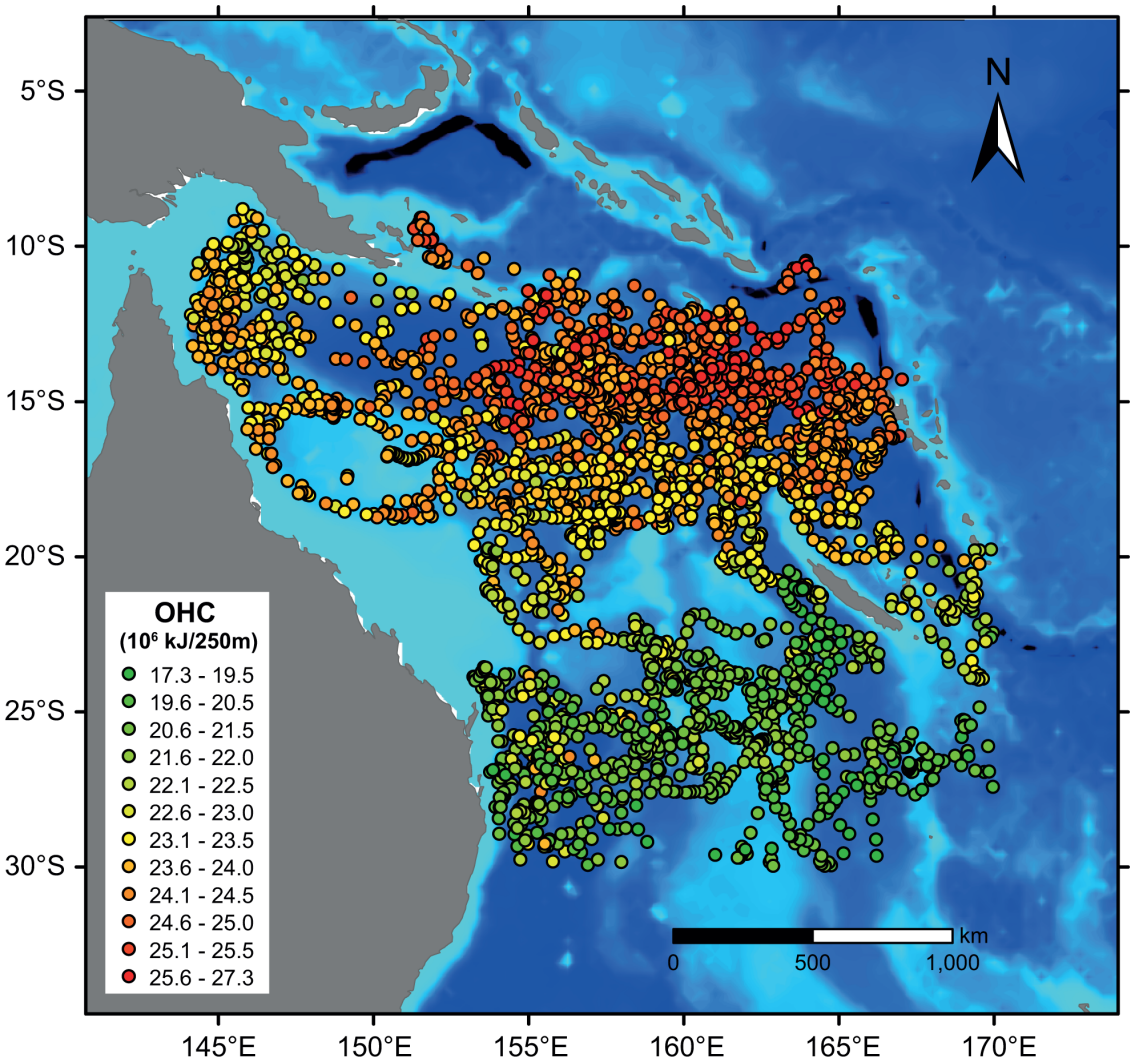
Geographical position of Argo floats within the Coral Sea. The colour scheme of the Argo data is denoting ocean heat content (OHC, 10^6^ kJ/250 m), which was integrated to a depth of 250 m. Locations are only displayed for CTD profiles that encompass the entire top 250 m of water.

The influence of the dominant western boundary currents (EAC and NQC) on, for example, temperature are well documented, with cooler water moving northwards due to the NQC and warmer water southward with the EAC [21]. This is also clearly evident in the upmost 75 m temperature fields of CARS2009 (CSIRO Atlas of Regional Seas, v. 2009), a global high-resolution climatology of seasonal ocean water properties [22]. However, while the presence of the NQC is evident in the relatively low Argo SSTs (Figure 6) and ocean heat content (Figure 7) in the north-western Coral Sea, the impact by the EAC is not apparent in the Argo data.

The main reason for the NQC showing greater presence and influence compared to the EAC in the observational dataset is owed to the Coral Sea bathymetry: while there are numerous Argo floats that travelled northward (due to the NQC) through a comparatively deep (and narrow) channel (Townsville and Queensland troughs), floats initially moving southwards with the EAC tended to get stranded on the shallow Coral Sea Plateau. Therefore, there are few Argo data available that recorded properties of the EAC, including the southward stream of relatively warm water mass. Consequently, the same area is also very sparse in Argo-derived MLD data. Similarly, the impedance of navigation in shallow waters signifies that Argo float availability for MLD computation is very limited throughout the continental shelf and plateaus, resulting in the absence of Argo-derived MLD data in some regions (e.g. Great Barrier Reef lagoon) and MLD clustering elsewhere.

### Suitability of the Threshold Method

The statistical analysis of the MLDs and ILDs, derived from four different threshold values, is shown in Tables 1 and 2. In general, the MLDs (0.03 kg/m^3^) and ILDs (0.2°C) obtained using the threshold method with values proposed by de Boyer Montégut *et al*. [11] compare quite well (±5 m) with the reference MLDs and ILDs (MLD_ref_ and ILD_ref_). In the case of the MLD, however, a threshold value of 0.04 kg/m^3^ appears to be more accurate in determining the MLD within the Coral Sea (Table 2), based on the lowest bias (0.2 m), mean absolute difference (2.4 m) and rmse (5.3 m). These results are substantiated by the highest skill score (0.96) associated with the 0.04 kg/m^3^ threshold value.

**Table 1:** Comparison of ILD_ref_ with the depths obtained by four different threshold values.

	ILD_0.1_	ILD_0.15_	ILD_0.2_	ILD_0.25_
**mean_ad_ (m)**	2.83±0.20	3.16±0.18	3.94±0.16	5.09±0.19
**bias (m)**	0.11±0.22	2.01±0.19	3.59±0.16	4.91±0.19
**rmse (m)**	7.98	7.32	7.06	8.54
**skill score**	0.91	0.92	0.93	0.90

The mean of paired absolute differences (mean_ad_) and bias (mean difference) are listed with their associated standard error (standard deviation divided by 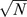, *N* = sample size).

**Table 2:** Comparison of MLD_ref_ with the depths obtained by four different threshold values.

	MLD_0.025_	MLD_0.03_	MLD_0.035_	MLD_0.04_
**mean_ad_ (m)**	3.20±0.21	2.63±0.18	2.42±0.15	2.40±0.13
**bias (m)**	−2.12±0.22	−1.10±0.19	−0.37±0.16	0.23±0.14
**rmse (m)**	8.41	7.20	5.94	5.33
**skill score**	0.90	0.92	0.95	0.96

The mean of paired absolute differences (mean_ad_) and bias (mean difference) are listed with their associated standard error (standard deviation divided by 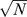, *N* = sample size).

The statistical results for the ILD are more complex (Table 1; Figure S1). The lowest rmse (7.1 m) is attributable to ΔT = 0.2°C, whereas the lowest bias (0.1 m) and mean absolute difference (2.8 m) are linked to ΔT = 0.1°C. There are advantages and disadvantages to both threshold values. Whereas threshold values in excess of 0.1°C have a tendency to overestimate the ILD (bias of 3.6 m for ΔT = 0.2°C), resulting in higher mean differences, ΔT = 0.1°C is less suitable for temperature profiles affected by restructuring (e.g. profiles modified by heavy rainfall or diurnal solar heating, with their impact exceeding 10 m). A threshold value of 0.1°C (and to a certain extent ΔT = 0.15°C) is predisposed to significantly underestimate the ILD when the isothermal layer is not well defined, consequently exhibiting a higher rmse (8.0 m). Overall, assuming that the temperature profiles are not thoroughly pre-screened, and profiles with poorly defined isothermal layers removed, a threshold value of 0.2°C for temperature profiles is the most appropriate for this region. This interpretation is also confirmed by the highest skill score (0.93) for the 0.2°C threshold value. A similar analysis of salinity profiles indicates that a threshold value of 0.02 psu most accurately predicts the isohaline layer depth.

As indicated earlier, factors which impact on the accuracy of the threshold method include the strong diurnal variability and heavy precipitation in localised areas. Their impact sporadically extends beyond the reference depth, forming a temporary mixing layer (region in which mixing is currently active [23]) which may be mistaken for the mixed layer. Even though a reference depth of 10 m is chosen to avoid the short-term variability, sometimes an underestimation of the MLD occurs due to diurnal solar heating or heavy precipitation affecting depths down to 15–20 m. For these profiles, the MLD (and ILD) is much more accurately determined by choosing 20 m (or 15 m, if appropriate) as the reference depth. This reference depth, however, cannot be chosen universally since summer mixed layers, at least, are frequently shallower than 20 m and can even be as shallow as ∼10 m, in which case the threshold method with a reference depth of 10 m would unavoidably result in an overestimation of the MLD and ILD.

### Temperature and Salinity Inversions

To obtain an accurate estimation of the MLD and ILD, threshold values for temperature (ΔT) and salinity (ΔS) have to take into account the possible presence of temperature and/or salinity inversions. Here, the ILD (and isohaline layer depth) was determined as the depth where temperature (salinity) has either decreased or increased by the given increment.

**Figure 8:**
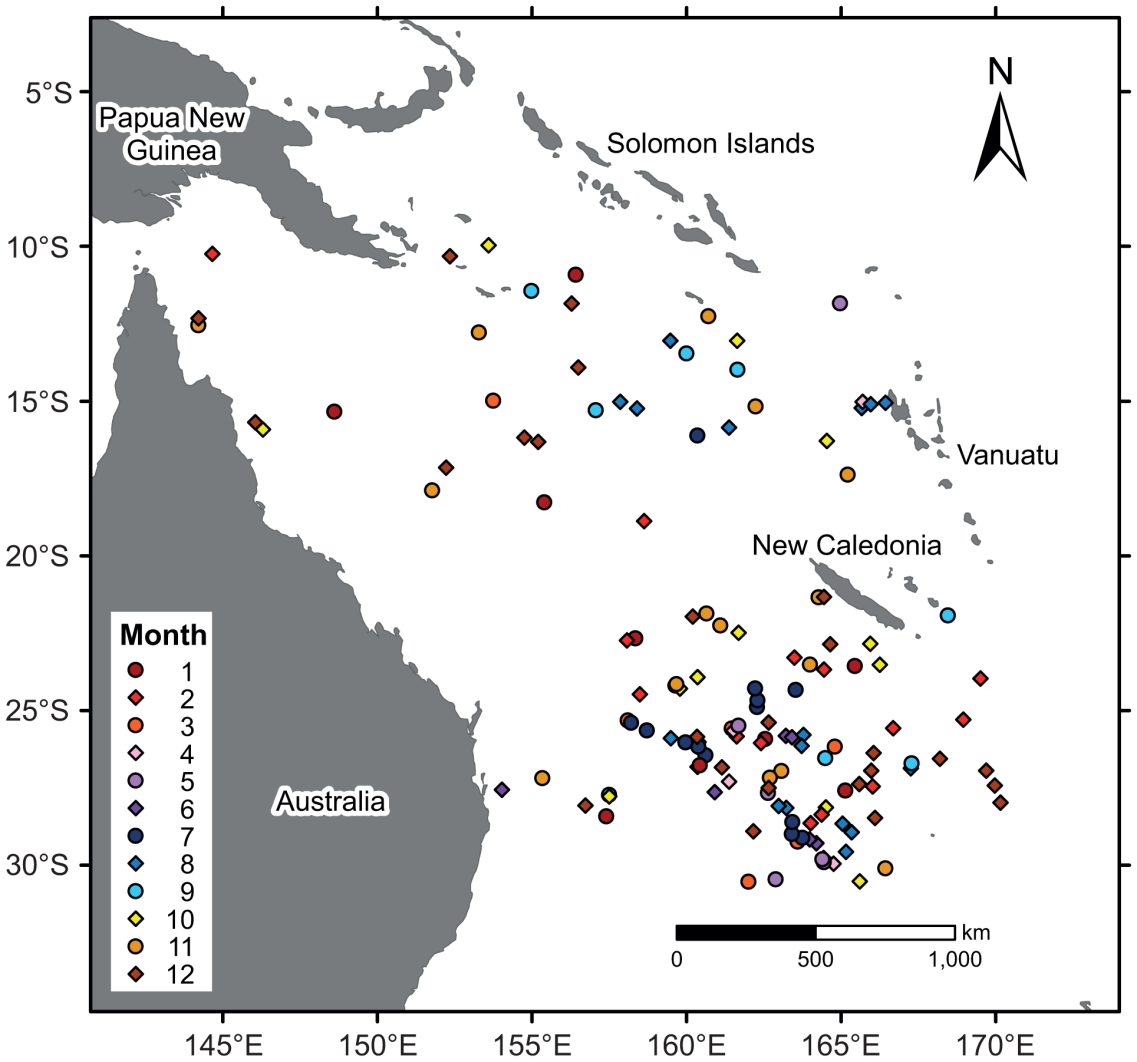
Locations within the Coral Sea where salinity inversions were observed.

While temperature inversions (increasing temperature with depth) appear to be very rare within the Coral Sea, salinity inversions (decreasing salinity with depth) are relatively common and occur year-round, although most of the salinity inversions are clustered within the south-eastern part of the Coral Sea (Figure 8). Salinity inversions in the south-east appear to be quite long-lasting as several Argo floats (mainly 5900572, 5900870, 5900871 and 5901511) displayed salinity inversions over prolonged and continuous periods. Compensated layers (i.e. layer of near-homogeneous density between a relatively shallow thermocline and a deeper halocline; cf. Fig. 10 in [11]) are, however, rarely observed. That is, the decline in density due to a salinity inversion is not enough to offset the increase in density due to the gradual decrease in temperature. In the presence of a compensated layer, an MLD estimation solely based on a density criterion would lead to an overestimation of the MLD. This is comparable to the inverse occurrence (barrier layer [24]), where an MLD inference based on temperature alone would also result in an MLD overestimation. Since a compensated layer occurrence is rare within the Coral Sea, MLD estimations based on density are appropriate. Conversely, MLD estimations based solely on temperature (i.e. ILD) have to be used cautiously due to the quite frequent occurrence (20%) of barrier layers within the Coral Sea, which result in ILDs that are significantly deeper than the MLDs.

**Figure 10:**
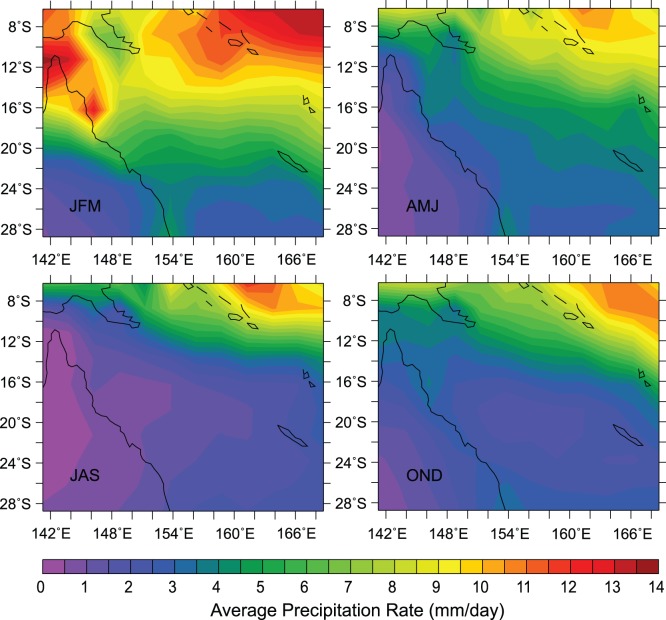
Rainfall seasonality. Seasonal mean precipitation rates (in mm/day) for January–March (top left), April–June (top right), July–September (bottom left) and October–December (bottom right). The CMAP Precipitation data is provided by the NOAA/OAR/ESRL PSD, Boulder, Colorado, USA (data freely accessible at http://www.cdc.noaa.gov/data/gridded/data.cmap.html).

### Barrier Layers

**Figure 9:**
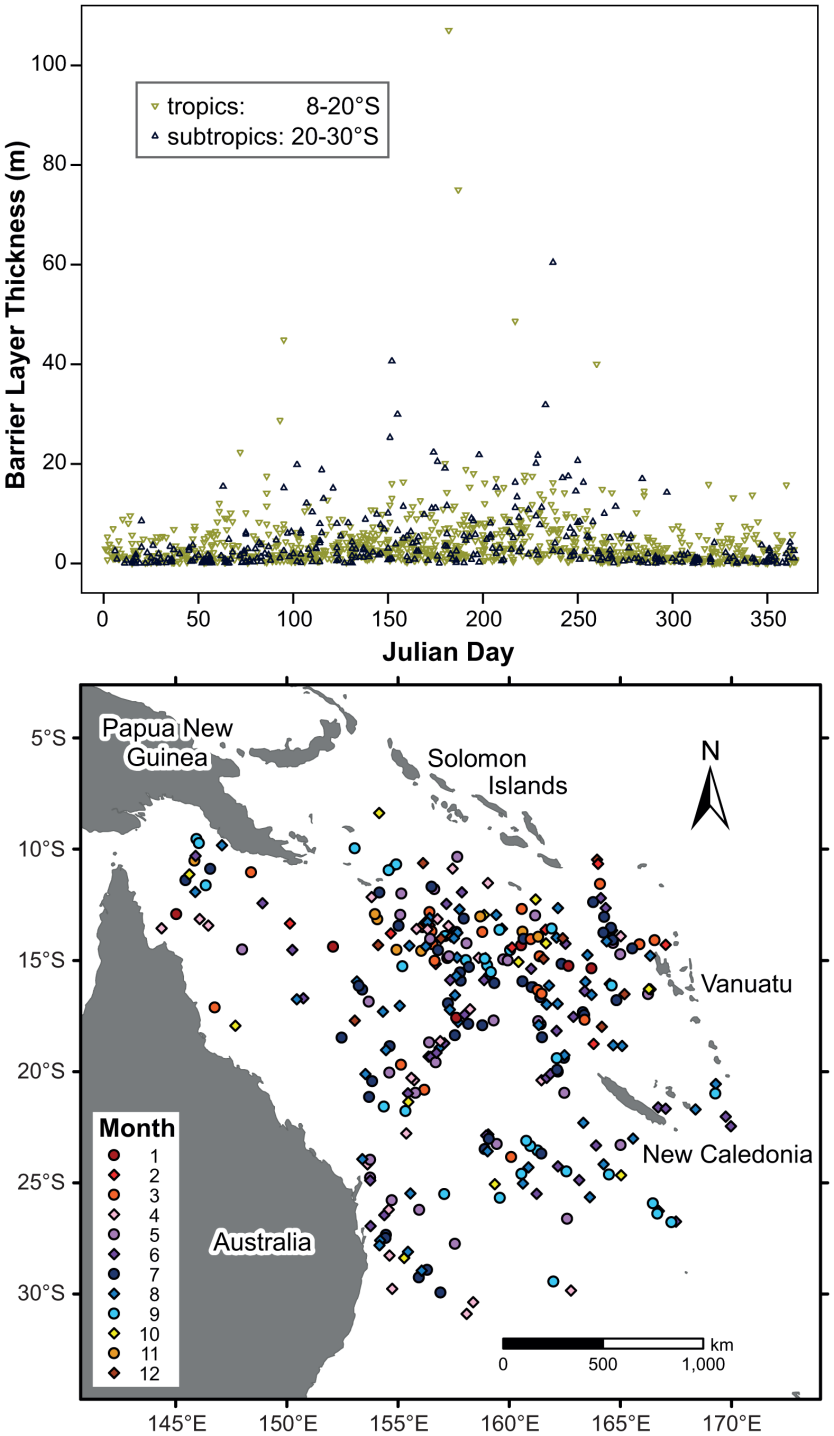
Barrier layer thickness (BLT) with respect to A) Julian day and B) geographical location. In figure B, only the BLTs exceeding 5 m are displayed.

Barrier layers are defined as the difference between the depth where the temperature has decreased by 0.2°C and the depth where the potential density has increased from the reference depth by a density threshold equivalent to the same temperature change (0.2°C) at constant salinity [25]. The vast majority (80%) of Argo profiles examined do not display signs of a significant barrier layer (>5 m) being present, such that there is no area with persistent barrier layers within the Coral Sea. With the potential exception of the south-western Coral Sea (south of 24°S and west of 157°E), where barrier layers are only observed during the cooler months, temporary barrier layer are not linked to a specific season (Figure 9a). Rather, they could develop throughout the Coral Sea any time of the year. There is a minor tendency towards the thickest barrier layers occurring during cooler months (March-September). This can be attributed to the, on average, deeper isothermal layers during austral winter, rather than to geographical location, SST, SSS or average temperature within the mixed layer.

In the north-eastern Coral Sea, the likelihood of a barrier layer formation is increased (Figure 9b), which was also noted in Mignot *et al*. ([26], their Figure 1). The increased barrier layer occurrence is linked to greater monsoonal activity and total average rainfall in that region compared to the remainder of the Coral Sea (Figure 10). Throughout the year, relatively high rainfall rates are noted in the north-eastern Coral Sea, frequently resulting in a lowering of salinity in the upper ocean. As a consequence of persistent precipitation, a shallower halocline may be induced, encouraging the formation of a temporary barrier layer.

### Tropical versus Subtropical MLD Seasonality

**Figure 11:**
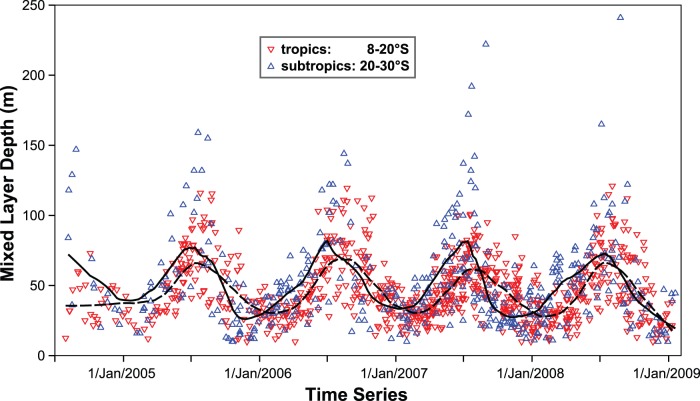
Evolution of the mixed layer depth (MLD_ref_) over a 4.5 year period. The line of best fit (solid-black for subtropics, dashed-grey for tropics) was created using the least-squared local regression (LOESS) method.

**Figure 12:**
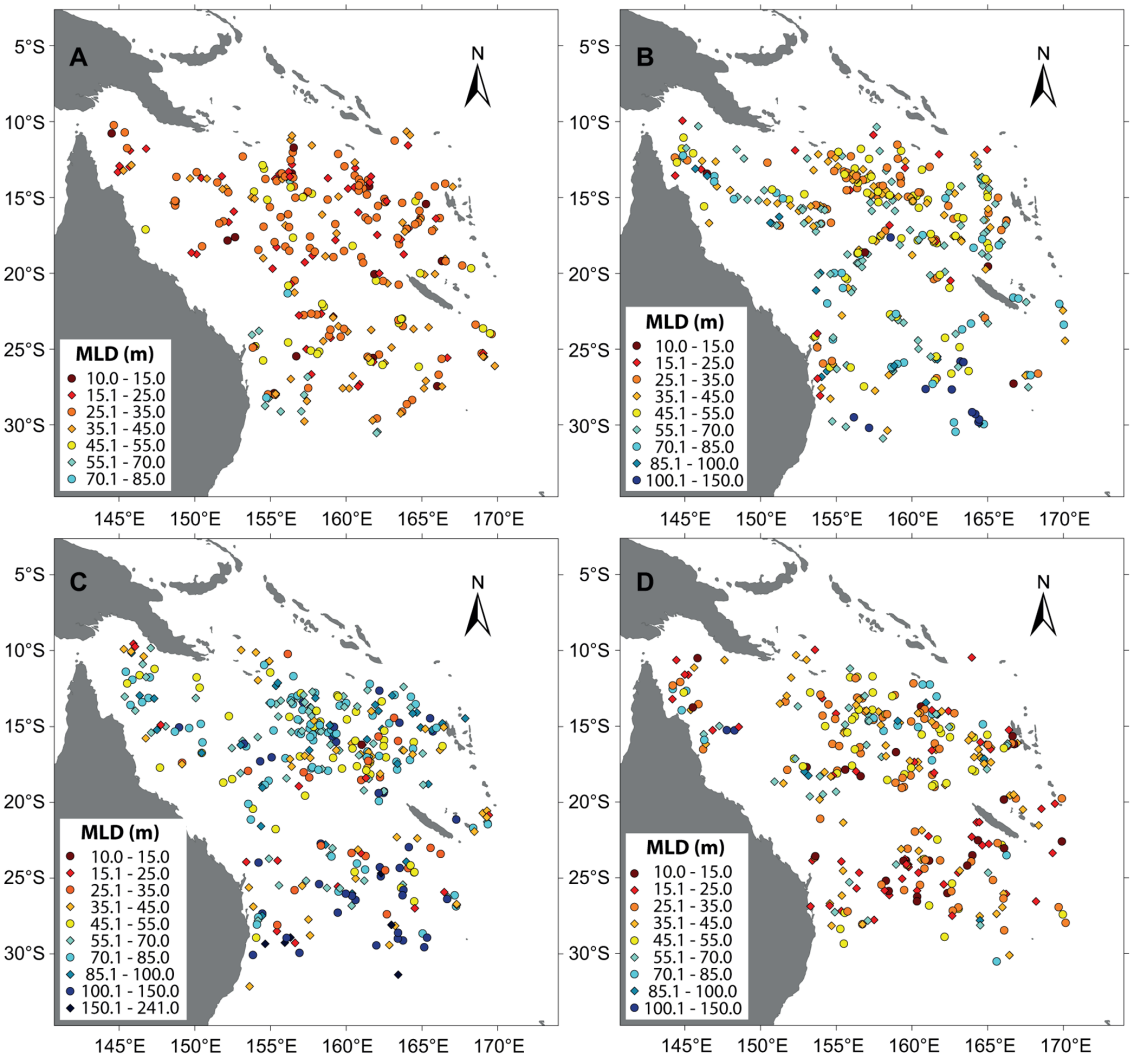
Seasonal variation of the mixed layer depth (MLD). MLD is shown for January–March (top left), April–June (top right), July–September (bottom left) and October–December (bottom right).

A clear seasonality in the MLD is evident throughout the Coral Sea. The MLD seasonality in higher latitudes is much more pronounced and tends to lead subtropical MLDs by a few weeks (Figure 11). Whereas summer mixed layers are shallow (10–50 m) throughout the Coral Sea, MLDs increase significantly towards the winter months in all regions (Figure 12). The deepening occurs due to a combination of a cooling upper ocean and elevated wind stress (predominantly south-easterly trade winds) during austral winter [27], both features facilitating increased mixing in the upper ocean. Conversely, mixed (and isothermal) layers are shallower in austral summer as a result of increased sea surface heating and relatively weak wind stress. In winter, tropical MLDs typically range from about 50 m to 100 m, whereas mixed layers in the higher Coral Sea latitudes commonly exceed 100 m and can occasionally surpass 200 m.

The seasonal deepening towards austral winter is also evident in pre-Argo data (Figure 4b). While the mean observed MLDs agree closely between Argo- and WOD-derived MLDs from January to July, there is a trend evident towards comparatively shallow WOD-MLDs from August to December. This can be ascribed to a combination of scarcity of WOD CTD profiles deep enough to capture thicker mixed layers, and the relative paucity of WOD CTD profiles in general.

Regionally, the deepest mixed layers are located in the south-western Coral Sea (Figure 12). In addition, the same area also displays some of the deepest summer MLDs, while shallow summer mixed layers are also observed. A similarly high variability has also been noted by Condie and Dunn [13], who linked it to the changing position of the EAC. In contrast, however, their summer MLDs tend to be less than 40 m in the south-west, a depth which many south-western Argo mixed layers exceed.

### Conclusion

An evaluation of four different threshold values for both temperature and density profiles revealed the extent of suitability of the threshold method for the Coral Sea. Due to the general absence of compensating and barrier layers, the threshold method predicts the ILD (ΔT = ±0.2°C) and MLD (Δρ = 0.04 kg/m^3^) reasonably well in this region. Discrepancies mainly occur when short-term sea surface variability (e.g. diurnal heat flux or intense rainfall) extends beyond 10 m depth, or when the ILD and/or MLD are not well defined due to restructuring of the upper ocean.

Analysis of 7.5 years worth of CTD data derived from Argo floats displays a strong seasonality in the MLD throughout the Coral Sea. As a result of larger seasonal variation in temperature and wind stress, MLDs in higher latitudes exhibit a greater seasonality (10–150 m) compared to those of lower latitudes (10–90 m). Summer MLDs are typically relatively shallow (10–50 m) and homogeneous throughout the Coral Sea. Subsequently, MLDs are deepening during autumn, with the thickest seasonal mixed layers generally observed during July and August. MLDs are then shallowing again to return to the minimum values during summer.

Salinity inversions are mainly found in the south-east, a turbulent region affected by the eddy-rich Tasman front. Conversely, barrier layers are formed throughout the Coral Sea but are mainly associated with the north-eastern Coral Sea, a region characterised by high precipitation.

The Argo floats program represents a significant advancement for marine studies, procuring near-continuous CTD data within the Coral Sea since mid-2001. However, the suitability of Argo data for a study strongly depends on the region of interest and the dominant currents. There are few features or effects evident by the EAC due to the shallow continental shelf inhibiting movement of Argo floats along the path of the EAC, resulting in limited data availability. Similarly, no Argo data is available for the Great Barrier Reef lagoon, a region of high scientific interest, due to shallow bathymetry. Conversely, a relatively large Argo dataset is available in the north-western Coral Sea due to the clockwise moving NQC, which transports cooler water mass northwards.

## Supporting Information

Figure S1 **Correlation between MLD_ref_ (ILD_ref_) and MLDs (ILDs) derived with the threshold method.**
(EPS)

Table S1 **Mean and standard deviation for the reference and calculated a) ILDs and b) MLDs.**
(DOC)

Table S2 **Numerical values associated with the skill score (SS) for a) ILD and b) MLD, with the threshold-derived MLDs (ILDs) compared with MLD_ref_ (ILD_ref_).**
(DOC)

Text S1 **Calculation of the skill score.**
(DOC)
