# Correction: Mixed Layer Depth Seasonality within the Coral Sea Based on Argo Data

**DOI:** 10.1371/annotation/59fd5655-cbd0-43ef-b434-43b0dbfbbc09

**Published:** 2013-08-06

**Authors:** Jasmine B. D. Jaffrés

There was an error in the title. The correct title is: "Mixed Layer Depth Seasonality within the Coral Sea based on Argo Data." The correct citation is: Jaffrés JBD (2013) Mixed Layer Depth Seasonality within the Coral Sea Based on Argo Data. PLoS ONE 8(4): e60985. doi:10.1371/journal.pone.0060985. 

In addition, in the online version of the article, Figure 10, "Rainfall seasonality," does not appear. Figure 10 does appear in the PDF, which can be viewed here: http://www.plosone.org/article/fetchObject.action?uri=info%3Adoi%2F10.1371%2Fjournal.pone.0060985&representation=PDF.

