# Correction: Mixed Layer Depth Seasonality within the Coral Sea Based on Argo Data

**DOI:** 10.1371/annotation/2e977b77-62e1-45b1-82c1-577cd745b60b

**Published:** 2013-08-23

**Authors:** Jasmine B. D. Jaffrés

In the original correction for doi:10.1371/journal.pone.0060985, the word "based" in the article title was noted as being incorrectly capitalized. However, this correction to the capitalization was not correctly reflected in a correction to the citation. The correct citation is: Jaffrés JBD (2013) Mixed Layer Depth Seasonality within the Coral Sea based on Argo Data. PLoS ONE 8(4): e60985. doi:10.1371/journal.pone.0060985. In addition, the original correction incorrectly stated that Figure 10 was not visible in the online version of the article. Figure 10 is visible in the online version, but it and Figure 9 were accidentally switched in the online version. 

