# Supplementary material for: Mixed Layer Depth Seasonality within the Coral Sea Based on Argo Data
Source: PLoS One. 2013 Apr 11;8(4):e60985. doi: 10.1371/journal.pone.0060985 (PMC3623957; doi:10.1371/journal.pone.0060985)
Supplement: Table S1 — Mean and standard deviation for the reference and calculated a) ILDs and b) MLDs. (DOC) [file pone.0060985.s002.doc]

**Table S1. Mean and standard deviation for the reference and calculated a) ILDs and b) MLDs.**

| **a)** | **ILDs** | **ILD_ref_** | **ILD_0.1_** | **ILD_0.15_** | **ILD_0.2_** | **ILD_0.25_** |
| --- | --- | --- | --- | --- | --- | --- |
|  | **mean (m)** | 51.0 | 51.1 | 53.0 | 54.6 | 55.9 |
|  | **standard deviation (m)** | 26.7 | 26.8 | 27.1 | 27.8 | 28.2 |
|  |  |  |  |  |  |  |
| **b)** | **MLDs** | **MLD_ref_** | **MLD_0.025_** | **MLD_0.03_** | **MLD_0.035_** | **MLD_0.04_** |
|  | **mean (m)** | 49.1 | 47.0 | 48.0 | 48.8 | 49.4 |
|  | **standard deviation (m)** | 26.2 | 25.9 | 26.1 | 26.3 | 26.5 |
