# Supplementary material for: Mixed Layer Depth Seasonality within the Coral Sea Based on Argo Data
Source: PLoS One. 2013 Apr 11;8(4):e60985. doi: 10.1371/journal.pone.0060985 (PMC3623957; doi:10.1371/journal.pone.0060985)
Supplement: Table S2 — Numerical values associated with the skill score (SS) for a) ILD and b) MLD, with the threshold-derived MLDs (ILDs) compared with MLDref (ILDref). (DOC) [file pone.0060985.s003.doc]

**Table S2. Numerical values associated with the skill score (SS) for a) ILD and b) MLD, with the threshold-derived MLDs (ILDs) compared with MLD_ref_ (ILD_ref_).**

| **a)** |  | **ILD_0.1-ref_** | **ILD_0.15-ref_** | **ILD_0.2-ref_** | **ILD_0.25-ref_** |
| --- | --- | --- | --- | --- | --- |
|  | **s_fx_ (m^2^)** | 683.69 | 698.44 | 724.41 | 729.98 |
|  | **r** | 0.95 | 0.97 | 0.98 | 0.97 |
|  | **SS** | 0.91 | 0.92 | 0.93 | 0.90 |
|  |  |  |  |  |  |
| **b)** |  | **MLD_0.025-ref_** | **MLD_0.03-ref_** | **MLD_0.035-ref_** | **MLD_0.04-ref_** |
|  | **s_fx_ (m^2^)** | 644.67 | 657.28 | 669.31 | 677.57 |
|  | **r** | 0.95 | 0.96 | 0.97 | 0.98 |
|  | **SS** | 0.90 | 0.92 | 0.95 | 0.96 |
