# Supplementary material for: Mixed Layer Depth Seasonality within the Coral Sea Based on Argo Data
Source: PLoS One. 2013 Apr 11;8(4):e60985. doi: 10.1371/journal.pone.0060985 (PMC3623957; doi:10.1371/journal.pone.0060985)
Supplement: Text S1 — Calculation of the skill score. (DOC) [file pone.0060985.s004.doc]

**Supporting information, “Mixed Layer Depth Seasonality within the Coral Sea based on Argo data”**

**Jasmine B. D. Jaffrés**

**Text S1: Calculation of the skill score**

The skill score used in this paper was derived and previously described by Murphy [1]. Here, the associated equations and values (Tables S1 and S2) are presented:

The correlation coefficient (*r*) between the estimated (*f*) and reference (*x*) MLD is defined as the covariance (*s_fx_*) between the estimated and reference MLD divided by the product of their standard deviations:

where *s_f_* is the standard deviation for the estimated MLD and *s_x_* is the standard deviation for the reference MLD.

The skill score (*SS*) is obtained by:

where and are the means of the estimated and reference MLD, respectively. The reader is referred to Murphy [1] for details on how this equation was derived. Scatter plots of estimated and reference MLDs and ILDs are provided in Figure S1, concomitant with r^2^ and the skill score associated with the threshold-derived depths.

# References

1. Murphy AH (1988) Skill scores based on the mean square error and their relationships to the correlation coefficient. Mon Wea Rev 116: 2417-2424.
